# Early diagnosis of gestational trophoblastic neoplasia based on trajectory classification with compartment modeling

**DOI:** 10.1186/s12874-015-0106-y

**Published:** 2016-01-05

**Authors:** Claire Burny, Muriel Rabilloud, François Golfier, Jérôme Massardier, Touria Hajri, Anne-Marie Schott, Fabien Subtil

**Affiliations:** Service de Biostatistique, Hospices Civils de Lyon, 162 avenue Lacassagne, F-69003 Lyon, France; Université de Lyon, F-69000 Lyon, France; Université Lyon 1, F-69100 Villeurbanne, France; CNRS, UMR 5558, Laboratoire de Biométrie et Biologie Evolutive, Equipe Biostatistique-Santé, F-69100 Villeurbanne, France; Department of Gynaecological and Oncological Surgery-Obstetrics, Lyon Sud University Hospital, Lyon, France; French Trophoblastic Disease Reference Centre, Lyon Sud University Hospital, Lyon, France; Department of Obstetrics, University Hospital Femme-Mère-Enfant, Lyon, France; Pôle Information Médicale Evaluation Recherche, Equipe d’Accueil 4129, Hospices Civils de Lyon, Lyon, France

**Keywords:** Early diagnosis, Model-based classification, CEM algorithm, Compartment model, Longitudinal study

## Abstract

**Background:**

In randomized clinical trials or observational studies, it is common to collect biomarker values longitudinally on a cohort of individuals. The investigators may be interested in grouping individuals that share similar changes of biomarker values and use these groups for diagnosis or therapeutic purposes. However, most classical model-based classification methods rely mainly on empirical models such as splines or polynomials and do not reflect the physiological processes.

**Methods:**

A model-based classification method was developed for longitudinal biomarker measurements through a pharmacokinetic model that describes biomarker changes over time. The method is illustrated using data on human Chorionic Gonadotrophic Hormone measurements after curettage of hydatidiform moles.

**Results:**

The resulting classification was linked to the evolution toward gestational trophoblastic neoplasia and may be used as a tool for early diagnosis. The diagnostic accuracy of the pharmacokinetic model was more reproducible than the one of a purely mathematical model that did not take into account the biological processes.

**Conclusion:**

The use of pharmacokinetic models in model-based classification approaches can lead to clinically useful classifications.

**Electronic supplementary material:**

The online version of this article (doi:10.1186/s12874-015-0106-y) contains supplementary material, which is available to authorized users.

## Background

An increasing number of studies in clinical research and epidemiology are collecting repeated biomarker measurements during follow-ups of subjects with various conditions or diseases. These longitudinal measurements, referred to as trajectories, are often used for patient monitoring. Sometimes, changes in biomarker values over time may also have a diagnostic or prognostic value. One example is the monitoring of Prostate Specific Antigen (PSA) to diagnose relapse of prostate cancer [1]. Another example is the monitoring of creatinine phosphokinase to check the condition of kidney-transplant patients [2]. Frequently, establishing a diagnosis with these repeated measurements uses a threshold value or comparisons between successive values (e.g., three successive increases or decreases). However, due to biological variability, a single biomarker value, or even a small number of successive values, may not be sufficiently reliable to establish a diagnosis. The whole trajectory may contain more information regarding diagnosis than a limited section of this trajectory. Moreover, the trajectory modeling process removes irrelevant fluctuations and may provide reliable information for diagnosis [1].

The adequate modeling of longitudinal data in a group of individuals varies according to the type of heterogeneity considered and the goal of the study. Mixed-effect models assume that individual trajectories are distributed around a single mean trajectory and that variability between individuals is normally distributed. However, summing up a sample of trajectories into a single one is not appropriate when there are really different patterns of trajectories; i.e., trajectories of various levels or shapes. In this case, the variability between individuals is not normally distributed. The use of classification models allows identifying *G* different typical trajectories and classifying the subjects according to these typical latent trajectories. Each typical trajectory being associated with a single group, it is hoped that these groups are linked, for example, to the diagnosis or the prognosis of the disease of interest.

The classical unsupervised classification approaches search for a partition of the trajectories that maximizes a geometric inertia criterion based on a given distance metric; they require, among other conditions, that measurements be taken on a regular basis, which is not always possible. The use of model-based classification methods overcomes this difficulty [3]: the data are viewed as stemming from a model that is parametric over time and whose parameter values vary from one group to another. The observed individual trajectories are thus analyzed by fitting a model and then classifying the trajectories into the most compatible, though latent, groups identified by the model. The estimation of the model parameters and the classification of the trajectories are performed jointly.

To define the typical group trajectories, model-based clustering relies often on non-parametric methods over time [4], splines, polynomials, or wavelet-based functions [5]. These methods are essentially descriptive and do not reflect the underlying biological mechanisms whereas the progression toward a disease is often linked to disturbances of a biological system. Herein, these methods are called non-biological methods. One alternative to these non-biological models could be the compartment models commonly used in pharmacokinetics or pharmacodynamics [6]; they constitute a compromise between a true biological model and a purely descriptive model, and are called herein “biological” compartment models. Compartment models tell the changes in biomarker quantities in different theoretical compartments of the body from entry to elimination through hepatic and/or renal pathways. The exchanges between compartments may be modeled by differential equations. These equations are parameterized by a set of kinetic parameters and transfer constants, which may be interpreted in biological and physiological terms. As these models reflect the functioning of biological mechanisms, it is likely that the classification obtained will be more linked to diagnosis or prognosis than a classification obtained by a non-biological modeling.

The aim of the present article is the presentation of a method developed to classify and model individual trajectories by integrating knowledge about physiology --through biological compartment models-- in order to find groups that make biological sense. The illustrative example deals with measurements of human Chorionic Gonadotrophic hormone (hCG) in women having undergone curettage or suction to evacuate a hydatidiform mole, a benign placental tumor developed during pregnancy. These hCG measurements are used for early detection of women who are most likely to develop gestational trophoblastic neoplasia (GTN) [7]. A compartment classification model of hCG trajectories is proposed. The resulting classification is eventually compared with the current reference diagnostic criteria.

## Methods

### A semi-mechanistic model for hCG data

#### The data about hCG after curettage

The present study involved women registered to the French Trophoblastic Disease Reference Center (TDRC, Lyon, France) from January 1st, 2010 to December 31st, 2012, and who underwent curettage for hydatidiform mole [8]. After curettage, the women should be followed-up with weekly measurements of total hCG until undetectable levels, then during 2 or 3 weeks for partial moles, or monthly during 6 months for complete moles. Curettage is usually followed by a spontaneous total evacuation of the mole in the best-case scenario. However, up to 25 % of women keep persistent molar residuals after curettage [9], which may lead to GTN, and to trophoblastic gestational tumor in 15 % of cases.

The data come from a French registry. According to the current French law, an observational study that does not change routine management of patients does not need to be declared or submitted to the opinion of a research ethics board (Loi Huriet-Sérusclat 88–1138, 20 December 1988). However, informed consent was obtained from all participating subjects.

For the present analysis, the objective being an early diagnosis of GTN, the time-interval after curettage was restricted to 21 days. We kept for analysis only data on women who had at least two hCG measurements and who underwent no other curettage during these 21 days. Among the 1053 women thus kept, 155 (14.7 %) were diagnosed with GTN after 21 days according to the Federation of Gynecology and Obstetrics (FIGO) criteria considered here as a gold standard [10]; these women are qualified as diseased thereafter and opposed to the other non-diseased women.

**Fig. 1:**
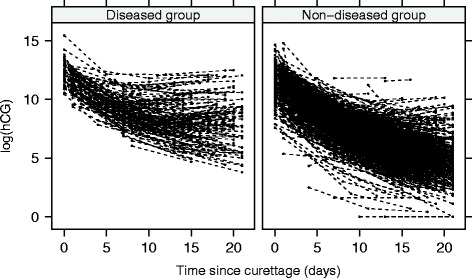
Trajectories of log-hCG values in diseased (*left*) and non-diseased (*right*) women after hydatidiform mole curettage

After curettage, there is usually an exponential two-phase decline of hCG, possibly followed by an increase in the diseased group (Fig. 1). Initial hCG level differences between women may depend on the initial size and vascularization of the mole, on some women characteristics [7], and even on measurement errors.

#### The biological compartment model

**Fig. 2:**
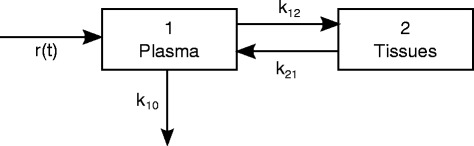
The two-compartment model for log-hCG values

After curettage, it is assumed that hCG hormone diffuses into the blood stream (the plasma compartment or Compartment 1) as a bolus, then hCG passes from plasma to tissues (Compartment 2). During this early fast phase, hCG plasma concentration declines until equilibrium is reached. This mutual exchange between plasma and tissues may be represented by transfer constants *k*_*12*_ and *k*_*21*_ (Fig. 2). During a second slower phase of elimination, hCG is filtered by the kidneys and captured by the liver, which are in equilibrium with plasma [11]. This elimination may be represented by an elimination constant *k*_*10*_. The above-mentioned bi-exponential decrease is consistent with a two-compartment model.

Moreover, there could be a residual hCG production (e.g., in case of GTN). Two hypotheses were made regarding this residual production: a constant hCG production over time, expressed as *r*(*t*) = *A*, or an increasing production over time due to the GTN growth, expressed as *r*(*t*) = *A × t*.

The biological compartment model shown in Fig. 2 can be converted into a system of ordinary differential equations (ODE):

1 $$ \left\{\begin{array}{l}\frac{d{\mu}_1(t)}{dt}=-\left({k}_{12}+{k}_{10}\right){\mu}_1(t)+{k}_{21}{\mu}_2(t)+r(t)\\ {}\frac{d{\mu}_2(t)}{dt}={k}_{12}{\mu}_1(t)-{k}_{21}{\mu}_2(t)\end{array}\right. $$

where *μ*_*1*_*(t)* and *μ*_*2*_*(t)* are the hCG concentrations at time *t* in plasma and tissues, respectively. We define **C** = [*A*, *k*_*12*_, *k*_*21*_, *k*_*10*_, *μ*_*10*_] as the set of parameters that characterizes the model and where *μ*_*10*_ = *μ*_*1*_*(0)* corresponds to the initial hCG plasma concentration. The initial concentration in the tissues was constrained to be zero, first because it was not observed, then to limit the number of parameters to estimate. All parameter values were constrained to be positive.

To assess the advantage of a biological compartment model, we tested a non-biological model. The hCG plasma concentration was modeled by a simple bi-exponential equation; thus, without taking into account the biological hypotheses:

2 $$ {\mu}_1(t)=c \exp \left(a\times t\right)+{c}_2 \exp \left({a}_2\times t\right) $$

We define **C'** = [*c*, *a*, *c*_*2*_, *a*_*2*_] as the set of parameters that characterizes Eq. 2. This bi-exponential model has been already used to model hCG values in women with low risks of gestational trophoblastic neoplasia treated with methotrexate [12]. It is also a common model for biomarkers in oncology; for example, for modeling the changes in prostate specific antigen after prostate cancer treatment [13, 14]. This model is more flexible than linear segments models that are also frequently used in oncology [15]. Other more sophisticated models could have been designed (e.g., tri-exponential models) but we focused on models with reasonable numbers of parameters because of the small number of measurements per subject (median of 3, min-max: 2–6).

The constraints put on the group-dependent parameters of the biological compartment model could not be put on the parameters of the non-biological model. In fact, in the biological model, the parameters can be grouped into parameters that reflect the normal functioning of the organism (*k*_*12*_, *k*_*21*_, *k*_*10*_) and do not change between groups and parameters that can be impacted by GTN (*A*, *μ*_*10*_) and that may change between groups. In the non-biological model, a residual hCG production due to GTN can impact both slope parameters (*a* and *a*_2_). Moreover, because the intercepts are correlated with the slopes, the residual hCG production can also impact *c* and *c*_2_ parameters. Hence, there is no reason for these parameters to be identical in the four groups. This is why no constraints were put on the group-dependent parameters of the non-biological model.

#### The measurement error model

After modeling the changes in biomarker concentration through fitting a biological compartment model, the *j*^th^ measurement of individual *i* at time *t*_*ij*_ (that is, *Y*_*ij*_) was linked to its predicted value *μ*_*1*_*(t*_*ij*_*)* at time *t*_*ij*_ by including a Gaussian measurement error with variance *σ*^*2*^ . To obtain normally distributed residuals per group, we performed a log-transformation of hCG concentrations.

### The model-based classification

#### Biological compartment model for G groups

It was assumed that there are *G* different groups of individuals, each group having its own set of kinetic parameters indexed by the number of the group (**C**_**g**_ for the *g*^th^ group). The clinical knowledge allowed fixing constraints to the parameters of the *G* groups. The possible development of a GTN, source of residual hCG production, was supposed not to interfere with the normal exchanges between compartments; hence, transfer constants *k*_*12*_ and *k*_*21*_ and the elimination constant *k*_*10*_ were forced to be equal in all *G* groups. The aim being distinguishing women with and without GTN, it was assumed that hCG reflects the presence of GTN only via the production term *A. A* was constrained to be zero in the group with the lowest levels of hCG (assuming no GTN in this group) and left free to vary in the other groups. The initial concentration *μ*_*10*_ was also considered group-dependent.

In the non-biological model, the vectors of parameters **C'**_**g**_ were left without constraints between the *G* groups.

Two different kinds of models were tested: models with a constant residual variance (*σ*^*2*^) and models with a residual variance for each *G* group (*σ*_*g*_^*2*^). Herein, **α**_**g**_ = [**C**_**g**_**,***σ*^*2*^_*g*_] denotes the parameters that characterizes the typical trajectory and the residual variance of the *g*^th^ group.

#### Classification algorithm

In classification modeling, the inter-individual variability is taken into consideration through identifying groups of women with similar trajectories as described by *G* sets of parameters **α**_**g**_. Let **z**_**i**_ = (*z*_*i1*_, …, *z*_*ig*_, …, *z*_*iG*_) where *z*_*ig*_ = 1 when individual *i* is assigned to group *g*, otherwise = 0. In a classification maximum likelihood approach (CML) [16], the aim is to estimate **z**_**i**_ and **α**_**g**_ by maximizing *ℓ*_*c*_, the augmented log-likelihood of the observations, also called classification log-likelihood:

3 $$ {\ell}_c\left(\mathbf{Y};\boldsymbol{\alpha}, \boldsymbol{\pi}, \mathbf{z}\right)=\sum_{i=1}^N\sum_{g=1}^G{z}_{ig} \log \left({\pi}_g{f}_g\left({\mathbf{Y}}_i,{\boldsymbol{\alpha}}_g\right)\right) $$

where **Y**_*i*_ is the vector of measurements of individual *i, π*_*g*_ the prior probability that any individual belongs to the *g*^th^ group --constrained by ${\displaystyle \sum_{g=1}^G{\pi}_g=1}$ -- and *f*_*g*_ the density function of **Y**_*i*_ in the *g*^th^ group governed by the set of parameters **α**_**g**_. Here, according to the proposed measurement error model, *f*_*g*_ is a multivariate Gaussian distribution with a diagonal variance-covariance matrix. This makes the implicit assumption that, conditionally to the group membership, all the residuals of a single individual are independent. Maximizing this function according to **z**, **α**, and ***π*** is very difficult. However, as proposed by Celeux and Govaert [17], an iterative algorithm called Classification Expectation Maximization (CEM) algorithm can be used. This algorithm is close to the Expectation Maximization (EM) algorithm [18]. The parameter values are first initialized. Then, the algorithm alternates between three steps. First, the posterior probabilities for a trajectory to belong to the different groups --given the parameter values that define the groups-- are calculated using Bayes Theorem (E-step). Each individual trajectory is then classified into the group for which it has the maximum posterior probability; this leads to the determination of the values of **z** (C-step). Then, parameters **α**_**g**_ and *π*_*g*_ of the groups are estimated by maximum likelihood according to the just estimated **z** values (M-step).

For the first iteration, it is also possible to provide an initial classification of individuals instead of parameter values. In this case, the algorithm begins with M-step.

The three steps (estimation, classification, and maximization) are repeated until a stopping criterion is satisfied. This criterion was a relative difference in *ℓ*_*c*_ between two successive iterations lower or equal to 10^−6^ [19].

There is though no guarantee that the CEM algorithm can reach the global maximum of the classification likelihood function because this depends on the initial values [17]. To help the CEM algorithm find the global maximum, it was initialized using the results from a stochastic version (called the Stochastic Expectation Maximization or SEM algorithm) run over 100 iterations [20]. The SEM algorithm differs from the CEM algorithm only by the fact that, during the C-step, the individual trajectories are not classified into groups that correspond to the maximum posterior probabilities but classified randomly according to the posterior probabilities of membership; i.e., according to a multinomial trial. The initialization of the CEM algorithm is thus provided with the best parameter estimates that correspond to the maximal value of *ℓ*_*c*_ over the 100 iterations of the SEM algorithm. The use of SEM and CEM algorithms successively was shown to avoid sub-optimal solutions [17]; this is because the random part of the SEM algorithm avoids being trapped in a local maximum.

The sensitivity of the results to the initial conditions as provided by the SEM was then tested through different initial allocations of individuals at the first iteration with the CEM algorithm. The classification obtained after changing the initial allocations of 10 individuals per group was compared with the original classification.

#### Adaptation of CEM algorithm to a system of ODE

Maximizing the classification log-likelihood requires the values of the predicted concentrations of the biomarker for any individual given **C**_**g**_. However, Eq. 1 is not a closed-form function of time; thus, obtaining *μ*_*1g*_*(t)* given **C**_**g**_ requires the solution of a system of ODE. For some simple systems of ODE, there are analytical solutions for each compartment, but this was not the case of the system analyzed here. Hence, numerical approximated solutions of the system of ODE were required.

Function lsoda of the deSolve package under R software [21] was used herein. It selects the appropriate multistep method of solving (Adam’s methods or backward differentiation formulas) and its order *n* depending on the nature of the problem (stiff or not) [22]. The absolute error tolerance for the approximated solutions was set to 10^−6^. During each iteration of the CEM algorithm, the solving of the system of ODE is necessary for both estimation and maximization steps.

The M-step involves non-linear regression through the maximization of the classification log-likelihood (Eq. 3). This was done with a Gauss-Newton algorithm that updates iteratively the parameter values without requiring the computation of the second derivatives of the parameters [23]. Optimization during the M-step was performed by function nls [24] in R software that allows also constrained optimization. Threshold 10^−6^ was used as convergence criterion for the Gauss-Newton algorithm.

Simulations were performed to test the validity of the proposed method in estimating the parameters of a system of ODE in a classification context. The simulation design and the results are presented in the Additional file 1.

#### Choice and validation of the classification model

Classification models require the choice of: i) a suitable model which best describes the biological phenomenon of interest through measurements; and, ii) a number of groups *G*.

Selecting the model and the number of groups was performed on a body of criteria. The total number of groups was fixed from two to four. Indeed, for a diagnostic purpose, at least two groups are necessary; however, more groups were considered to allow for heterogeneity between women. Classical statistical criteria were considered to select the models: the log-likelihood and the penalized log-likelihood; namely, the Bayesian information criteria (BIC), the Akaike information criterion (AIC), and the integrated classification likelihood (ICL) [25]. The fitting of the models was also checked graphically. The obtained classification was compared with the true status regarding GTN as per the FIGO criteria. In case of more than two groups, the group with the highest trajectory level was considered as predictive of GTN and the other groups were considered as predictive of absence of GTN. The diagnostic accuracy features (sensitivity, specificity, positive and negative predictive values –PPV and NPV–, percentage of well-classified individuals) were calculated and also used for the choice of the model and the number of groups. A four-fold cross-validation [26] was performed on the retained models to reduce the optimism in the estimation of these features of diagnostic accuracy. The cross-validation was stratified on the true status of the women.

In our application, various models were considered according to the number of groups, the residual hCG production hypotheses (constant or increasing over time), and the variance of the residuals (constant or different in each group).

## Results

Table 1 shows the statistical performance criteria and the diagnostic accuracy features of the models tested after initialization through SEM. The log-likelihood, the penalized log-likelihood criteria (BIC, AIC, and ICL), and the global percentage of correctly classified women improved along with the number of groups whatever the constraint on the residual variance. This confirmed the presence of heterogeneity between women with residual hCG production after curettage. Hence, models with four groups were preferred. Models with a constant residual variance between groups tended to give higher PPVs than models with different residual variances; however, the former tended to give smaller NPVs. Models with constant residual variance provided also better specificities than models with varying residual variance because they classified fewer women in the upper group of hCG level, which was considered predictive of GTN. Regarding parsimony, the models that considered different residual variances between groups did not improve the classification.

**Table 1:** Statistical criteria and diagnostic accuracy of the fitted models

Variance^a^	G	Model^b^	Log- likelihood	BIC	AIC	ICL	Sensitivity (%)	Specificity (%)	PPV (%)	NPV (%)	Classification rate (%)^c^	Group sizes^d^
σ^2^	4	A	−4861	9836	9751	9656	36.1	96.8	65.9	89.8	87.8	110-460-398-85
σ^2^	4	A *×* t	−4932	9997	9892	9794	43.9	94.9	59.7	90.7	87.4	112-439-388-114
σ^2^	3	A	−5105	10300	10233	10174	55.5	91.3	52.4	92.2	86.0	256-633-164
σ^2^	3	A *×* t	−5117	10324	10257	10196	56.8	90.3	50.3	92.4	85.4	245-633-175
σ^2^	2	A	−5517	11098	11050	11026	76.8	73.7	33.5	94.8	74.2	698-355
σ^2^	2	A *×* t	−5509	11084	11035	11011	74.2	77.7	36.5	94.6	77.2	738-315
σ_g_^2^	4	A	−4552	9241	9138	8886	65.2	86.5	45.5	93.5	83.4	153-324-354-222
σ_g_^2^	4	A *×* t	−4699	9535	9432	9236	61.3	79.0	33.5	92.2	76.4	329-379-61-329
σ_g_^2^	3	A	−4924	9954	9875	9749	74.2	79.1	38.0	94.7	78.3	292-459-303
σ_g_^2^	3	A *×* t	−4923	9951	9872	9748	71.6	80.4	38.7	94.3	79.1	298-468-287
σ_g_^2^	2	A	−5519	11110	11055	11032	82.6	64.7	28.8	95.6	67.3	608-445
σ_g_^2^	2	A *×* t	−5511	11095	11040	11020	76.8	73.9	33.7	94.9	74.4	700-353
σ^2^	4	Non-biological	−4830	9823	9701	9632	41.9	95.9	63.7	90.5	87.9	111-445-395-102

^a^ *σ* for models with constant residual variances and *σ*_g_ for models with different residual variance between groups.^b^Type of residual hCG production: constant (r(t) = A), proportional to time (r(t) = A *×* t), and non-biological model. ^c^Percentage of well-classified subjects. ^d^Number of subjects per group from the lowest to the highest hCG trajectory group

For early detection of GTN, the best compromise between model fit and good features of diagnostic accuracy was reached with two models both with four groups (summarizing distinct patterns of hCG concentration changes) and a constant variance (yielding the highest PPVs) but one with a constant and the other with an increasing residual hCG production over time.

**Fig. 3:**
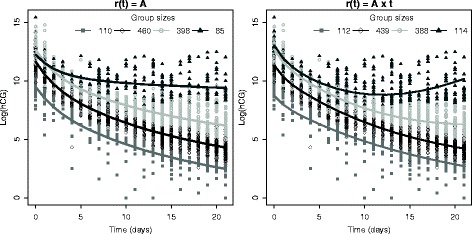
Observed measurements and typical trajectories with the two biological compartment models. Detailed-legend: The *solid lines* represent the typical trajectories. The *left panel* corresponds to the model with increasing residual hCG production and the *right panel* to the model with constant residual hCG production

Figure 3 shows the per-group typical trajectories with each of these models. The typical trajectory of the upper group is characterized either by an unchanged (Fig. 3, left) or an increasing (Fig. 3, right) hCG level after an initial exponential decrease. In both models, there were high NPVs (89.8 and 90.7 %) indicating that the physician may be nearly sure of the absence of GTN when a women is classified into one of the three lower groups. The PPVs of the highest group were respectively 65.9 and 59.7 % which indicates that the model with a constant (vs. increasing) residual hCG production would give more reliable positive tests. However, the latter model had a higher sensitivity (43.9 vs. 36.1 %).

We then tested the sensitivity of the classification to the initial conditions of the CEM algorithm by changing three times the initial classification with both selected models. With both models, the sensitivity analysis did not converge exactly to the same solution; however, only three women at the most changed groups without noticeable changes in the log-likelihood values.

The diagnostic accuracy features of both models were re-estimated by a 4-fold cross-validation. The median results over the four validations are shown in Table 2. The cross-validated diagnostic accuracy features were close to the original ones. Hence, there was little or no optimism in the estimation of the diagnostic performances.

**Table 2:** Median values of the cross-validated diagnostic accuracy features

Model^a^	Sensitivity	Specificity	PPV	NPV	Classification rate (%)
	(%)	(%)	(%)	(%)	
A	34.9	97.1	67.0	89.6	87.9
A *×* t	44.6	94.8	59.4	90.8	87.4
Non-biological	24.6	93.8	41.6	87.9	83.6

^a^Type of residual hCG production: constant (r(t) = A), proportional to time (r(t) = A *×* t), or non-biological model

We also evaluated the non-biological bi-exponential model (Eq. 2) assuming four groups. The diagnostic accuracy features were similar between each of the two biological compartment models and the non-biological model. The statistical criteria BIC and ICL were even better with the non-biological model. However, considering the results of the 4-fold cross validation shown in Table 2, it appeared that the non-biological model was worse in terms of sensitivity; there was an absolute loss of more than 19 % sensitivity with regard to the model with an increasing residual hCG production. This means a loss of ability to detect diseased women. The absolute difference by more than 25 % PPV between the non-biological model and the model with a constant residual hCG production indicated that using the PPVs relative to the non-biological model would increase the probability of wrong treatments.

Finally, the results of the simulations performed to test the validity of the proposed method in estimating the parameters are given in the Additional file 1. The global classification error rate was generally acceptable (close to 7 % for a realistic residual variance) and increased with the increase of the residual variance and the decrease of the sample size. The relative bias was generally acceptable (lower than 5 %).

## Discussion

In the present study, a model-based classification was performed with the CEM algorithm to identify typical trajectories associated with groups of individuals and assign each individual to one of these groups. Our method is applicable in presence or absence of explicit solution for the system of ordinary differential equations used to describe the trajectories. The small simulation study showed that the algorithm proposed for data classification and parameter estimation seems valid.

To our knowledge, this is one of the first applications of a model-based classification inspired by a biological model. Unlike a purely non-biological model, such as the bi-exponential model used here, a biological compartment model reflects biological facts. Indeed, in the present application, the non-biological model had the best BIC and ICL values; however, according to the cross-validation results, this model was not as satisfactory as the biological model in predicting the disease in new subjects. Contrarily to the biological compartment model, the non-biological model did not take into account the biological processes that underlie the changes in hCG level, which may explain the poor reproducibility of the features of its diagnostic accuracy. Moreover, the non-biological model might overfit the data due to the higher number of parameters compared to the biological model (20 vs. 17); this induces optimism and poor reproducibility [27]. The constraints put on the group-dependent parameters of the biological compartment model might have improved the diagnostic ability of the classification; in the non-biological model, such constraints were not introduced because they were difficult to determine and justify; indeed, in this model, the parameters have no biological meaning.

Considering trajectory heterogeneity between individuals has been already proposed through the use of a non-linear mixed-effects modeling, which is common in pharmacokinetics or pharmacodynamics [28], though mixed models do not lead to a classification of trajectories. Taking into account the natural heterogeneity may be also achieved through another approach with a finite mixture model, often called growth mixture model [29–31]. This approach assumes that each individual trajectory is a mixture of *G* typical trajectories with weights that vary from one individual trajectory to another. However, these typical trajectories may not be clinically relevant; they are only statistical constructs that achieve a close modeling of the data. Besides, mixture models do not assign each individual trajectory to a given group during the process of parameter estimation: each individual trajectory has only a probability (or degree of membership) of belonging to a typical trajectory. Hence, it is not always possible to give a clinical meaning to a typical trajectory when it does not represent a set of individual trajectories. For diagnostic purposes, the aim is to classify the individual trajectories into a limited number of groups; thus, an alternative to mixture models is necessary.

In the method proposed here, the predicted trajectory for a given individual is the typical trajectory of the group to which this individual belongs. However, even within a group, an individual trajectory may deviate from the typical trajectory. This may be taken into account by adding a random effect within each group, combining thus a categorical variable (i.e., label *g* of the group) with intra-group heterogeneity. Within the context of mixture modeling, this approach would be similar to the one proposed by Muthén and Shedden [32] and would take into account the correlation between the observations of each individual. For the time being, these observations are considered independent given the group. Adding the random effect can be implemented with Monolix, the standard software for mixed-effect modeling in a population context that uses a stochastic approximation of EM (SAEM) algorithm to simulate the unobserved individual parameters [33]. The implementation of this approach needs further investigations.

Here, we have chosen for the data a Gaussian distribution per group, though various other distributions could have been used. More research is needed to work with more flexible distributions according to the assumption on the distribution of the residuals [34].

CART or random forest methods could be alternatives to the proposed classification method. For example, the regression trees approach has been extended to longitudinal data [35]. However, some of the implementation of these methods require the same number of measurements per subject and this was not the case in our example. Moreover, these methods require to partition the time of follow-up. In the case of a marker with very quick changes, like hCG, the results might be sensitive to this partition. This is why we used an approach with a parametric modeling of marker changes over time.

The present work used only the log-likelihood, BIC, AIC, and ICL as statistical criteria for setting the number of groups. Various other statistical criteria have been proposed for this purpose [36]. Some of them have been compared in previous publications [37, 38] but there is no general consensus on the best criterion. Here, the use of different statistical criteria led to the same model. One general consensus is, however, that the number of groups should not be chosen only on the basis of statistical criteria but also on the notion of clinical relevance [39]. Some articles or books give application guidance on choosing the number of groups [36, 40, 41]. Here, the number of groups was chosen not only on statistical criteria but also on the diagnostic accuracy features of the models, and on the relevance of the model regarding the number of subjects per group.

In the present study, the maximum number of groups was fixed to four. In fact, a model with constant hCG production over time involving five groups led to a lower BIC than the model with four groups (9413 vs. 9836) but included a group with only 24 subjects (data not shown). With such a small group, the model was unstable and the results would have lacked reproducibility when tested by cross-validation. Regarding the computation time, more than 18 and 24 h were required for the two selected models *r(t) = A* and *r(t) = A × t*, respectively (including the SEM initialization pre-step) on an Intel Core i5. With these models, more than 95 and 98 % of the computation time was spent on the SEM step that searched for the optimal initial parameter values. It is especially necessary to prevent, as much as possible, the CEM algorithm from finding local optimum estimates because the log-likelihood has a complex expression with multiple optima. Reducing the number of SEM repetitions might not be conceivable; instead, another optimization algorithm may be used to increase the speed of convergence; e.g., the Levenberg-Marquardt algorithm [42] which differs from the Gauss-Newton algorithm only by the descent direction.

Regarding early detection of GTN, depending on the chosen model, the women in the highest group had 59.7 or 65.9 % probability of developing GTN. This group identified 34.9 or 44.6 % of the diseased subjects as per FIGO criteria. These percentages are obviously too low; however, in this group, the model-based classification up to day 21 would diagnoses GTN at a median of 18 days with the constant residual hCG production model and a median of 20 days with the varying residual hCG production model before the diagnosis on the current FIGO criteria.

The attainment of relatively high NPVs (close to 90 %) suggested the two selected models might help identifying women who do not require aggressive or toxic therapies, which is an important clinical benefit.

In fine, choosing between the two selected models (constant or increasing residual hCG production over time) should not be solely based on statistical criteria but consider also the whole clinical context. This goes however beyond the scope of the present article.

A three-level GTN risk classification may be also considered: i) women in the lower group (Fig. 3) with a low GTN risk whose follow-up may be stopped at 21 days; ii) women in the two middle groups with an intermediate GTN risk who require an active follow-up for more than 21 days before taking the decision to treat; and, iii) women in the higher group with a high GTN risk who might be treated right after the 21 days of follow-up. Note that the lower typical trajectory is characterized by low initial hCG values; hence, the women in this group might be identified by the first hCG value [7].

One should keep in mind that the diagnostic accuracy features of our method may have been underestimated because of the reliance on an imperfect gold standard (The FIGO criteria). A more reliable evaluation would have been obtained if histological proofs had been available for all women.

In the present study, we did not consider the initial type of mole because, despite pathological and clinical differences, the management of women with complete or partial mole is the same [7]. However, this distinction might be beneficial because these two types do not have the same risks of developing complications: 0.5 to 9.9 % vs. 20 %, respectively [9]. Further investigations on a larger sample of sujects or on the complete-mole part of the dataset are needed. A polytomous logistic regression could be coupled with the classification model to include predictors of group membership, such as the type of mole.

The present method may be applied to other clinical classification problems that involve longitudinal measurements and comply with compartment modeling. Further applications are needed to establish whether the present method that relies on compartment models leads always to more clinically useful classifications and more reproducible diagnostic accuracies than methods that rely on non-biological models.

## Conclusions

The use of a compartment model may give a clinical sense to specific trajectory classifications and hence be useful for diagnostic, prognostic, or therapeutic purposes. In the example of the hydatidiform mole, the application of the method to the hCG measurements led to earlier diagnoses of GTN than the classical FIGO criteria and to more reproducible results than those obtained with purely mathematical models. Applications on other examples are undeway.

## Additional file

Additional file 1:
**Simulation design and results.** (PDF 101 kb)

## References

[1] Subtil F, Rabilloud M (2010). Robust non-linear mixed modelling of longitudinal PSA levels after prostate cancer treatment. Stat Med.

[2] Josephson MA (2011). Monitoring and managing graft health in the kidney transplant recipient. Clin J Am Soc Nephrol.

[3] Fraley C, Raftery AE (2002). Model-based clustering, discriminant analysis, and density estimation. J Am Stat Assoc.

[4] Genolini C, Falissard B (2011). KmL: a package to cluster longitudinal data. Comput Methods Programs Biomed.

[5] Giacofci M, Lambert-Lacroix S, Marot G, Picard F (2013). Wavelet-based clustering for mixed-effects functional models in high dimension. Biometrics.

[6] Sheiner LB, Steimer JL (2000). Pharmacokinetic/pharmacodynamic modeling in drug development. Annu Rev Pharmacol Toxicol.

[7] Soper JT (2006). Gestational trophoblastic disease. Obstet Gynecol.

[8] Schmitt C, Doret M, Massardier J, Hajri T, Schott AM, Raudrant D (2013). Risk of gestational trophoblastic neoplasia after hCG normalisation according to hydatidiform mole type. Gynecol Oncol.

[9] Wolfberg AJ, Feltmate C, Goldstein DP, Berkowitz RS, Lieberman E (2004). Low risk of relapse after achieving undetectable HCG levels in women with complete molar pregnancy. Obstet Gynecol.

[10] Kohorn EI (2001). The new FIGO 2000 staging and risk factor scoring system for gestational trophoblastic disease: Description and critical assessment. Int J Gynecol Cancer.

[11] Schoeberl MR (2007). A model for the behavior of beta-hCG after evacuation of hydatidiform moles. Gynecol Oncol.

[12] You B, Pollet-Villard M, Fronton L, Labrousse C, Schott AM, Hajri T (2010). Predictive values of hCG clearance for risk of methotrexate resistance in low-risk gestational trophoblastic neoplasias. Ann Oncol.

[13] Zagars GK, Pollack A (1993). The fall and rise of prostate-specific antigen. Kinetics of serum prostate-specific antigen levels after radiation therapy for prostate cancer. Cancer.

[14] Taylor JM, Yu M, Sandler HM (2005). Individualized predictions of disease progression following radiation therapy for prostate cancer. J Clin Oncol.

[15] Bellera CA, Hanley JA, Joseph L, Albertsen PC (2008). Hierarchical changepoint models for biochemical markers illustrated by tracking postradiotherapy prostate-specific antigen series in men with prostate cancer. Ann Epidemiol.

[16] Symons MJ (1981). Clustering Criteria and Multivariate Normal Mixtures. Biometrics.

[17] Celeux G, Govaert G (1992). A classification EM algorithm for clustering and two stochastic versions. Comput Stat Data Anal.

[18] Dempster AP, Laird NM, Rubin DB (1977). Maximum likelihood from incomplete data via the EM algorithm. J R Stat Soc Series B Stat Methodol.

[19] Biernacki C, Celeux G, Govaert G, Langrognet F (2006). Model-based cluster and discriminant analysis with the MIXMOD software. Comput Stat Data Anal.

[20] Biernacki C, Celeux G, Govaert G (2003). Choosing starting values for the EM algorithm for getting the highest likelihood in multivariate Gaussian mixture models. Comput Stat Data Anal.

[21] R Development Core Team (2012). R: A language and environment for statistical computing.

[22] Petzold L (1983). Automatic Selection of Methods for Solving Stiff and Nonstiff Systems of Ordinary Differential Equations. SIAM J Sci Comput.

[23] Pereyra V (1967). Iterative Methods for Solving Nonlinear Least Squares Problems. SIAM J Numer Anal.

[24] Bates DM, Watts DG (2007). Nonlinear Regression Analysis and Its Applications.

[25] Biernacki C, Celeux G, Govaert G (2000). Assessing a mixture model for clustering with the integrated completed likelihood. IEEE Trans Pattern Anal Mach Intell.

[26] Stone M (1974). Cross-Validatory Choice and Assessment of Statistical Predictions. J R Stat Soc Series B Stat Methodol.

[27] Smith GC, Seaman SR, Wood AM, Royston P, White IR (2014). Correcting for optimistic prediction in small data sets. Am J Epidemiol.

[28] Tornøe CW, Agersø H, Jonsson EN, Madsen H, Nielsen HA (2004). Non-linear mixed-effects pharmacokinetic/pharmacodynamic modelling in NLME using differential equations. Comput Methods Programs Biomed.

[29] Muthén B, Kaplan D (2004). Latent variable analysis: Growth mixture modeling and related techniques for longitudinal data. The SAGE Handbook of Quantitative Methodology for the Social Sciences.

[30] Proust-Lima C, Letenneur L, Jacqmin-Gadda H (2007). A nonlinear latent class model for joint analysis of multivariate longitudinal data and a binary outcome. Stat Med.

[31] Neelon B, Swamy GK, Burgette LF, Miranda ML (2011). A Bayesian growth mixture model to examine maternal hypertension and birth outcomes. Stat Med.

[32] Muthén B, Shedden K (1999). Finite mixture modeling with mixture outcomes using the EM algorithm. Biometrics.

[33] Lavielle M, Mbogning C (2013). An improved SAEM algorithm for maximum likelihood estimation in mixtures of non linear mixed effects models. Stat Comput.

[34] Lu X, Huang Y (2014). Bayesian analysis of nonlinear mixed-effects mixture models for longitudinal data with heterogeneity and skewness. Stat Med.

[35] Loh W-Y, Zheng W (2013). Regression trees for longitudinal and multiresponse data. Ann Appl Stat.

[36] Nagin DS (2005). Group-Based Modeling of Development.

[37] Milligan GW, Cooper MC (1985). An examination of procedures for determining the number of clusters in a data set. Psychometrika.

[38] Shim Y, Chung J, Choi I (2005). A comparison study of cluster validity indices using a nonhierarchical clustering algorithm. Proceedings of the Computational Intelligence for Modelling, Control and Automation, 2005 and International Conference on Intelligent Agents, Web Technologies and Internet Commerce.

[39] Everitt B, Landau S, Leese M (2001). Cluster Analysis.

[40] Nagin DS, Odgers CL (2010). Group-based trajectory modeling in clinical research. Annu Rev Clin Psychol.

[41] Nylund KL, Asparouhov T, Muthén BO (2007). Deciding on the number of classes in latent class analysis and growth mixture modeling: a Monte Carlo simulation study. Struct Equ Modeling.

[42] Marquardt DW (1963). An Algorithm for Least-Squares Estimation of Nonlinear Parameters. SIAM J Numer Anal.

