# Supplementary material for: Early diagnosis of gestational trophoblastic neoplasia based on trajectory classification with compartment modeling
Source: BMC Med Res Methodol. 2016 Jan 5;16:3. doi: 10.1186/s12874-015-0106-y (PMC4702411; doi:10.1186/s12874-015-0106-y)
Supplement: Additional file 1: — Simulation design and results. (PDF 101 kb) [file 12874_2015_106_MOESM1_ESM.pdf]

Early diagnosis of gestational trophoblastic neoplasia based on trajectory classification with compartment modeling

Simulation design and results

Simulation design

The data were simulated so that they look like the data analyzed in the hCG study. The mean trajectory of a given group was the mean trajectory estimated in the hCG study by the model that considered: four groups, constant variance, and increasing residual hCG production over time. Only the three upper groups were kept for the simulation study. The total number of patients was either 1000 (three groups of 100, 500, and 400 patients; i.e., close to the numbers of patients estimated in the hCG study), 200 (20, 100, and 80 patients), or 100 (10, 50, and 40 patients). Hence, only the total number of patients was changed but not their repartition between the groups.

The number and time points of the measurements per patient were obtained by sampling with replacement from the number and time points of the measurements made in the patients of the hCG study. The residual variance was fixed to 0.747 (the value estimated in the hCG study) or to 1.121 (1.5 times the latter value).

For each number of patients per group and residual variance, data were simulated 100 times and the same model as the one used to simulate the data was adjusted to the data to identify the three groups. The global classification error rate was calculated, as well as the classification error rate per group. For each parameter, the mean bias, the standard deviance, and the root mean squared error (RMSE) were calculated.

Results

The classification errors are shown in Table A1. The global classification error rate was close to 7% for a residual variance of 0.747 and close to 17% for a residual variance of 1.121. This rate did not change much according to the total number of patients. The per-group classification error rates were the highest in the first group (the one with the smaller number of patients) and increased together with the residual variance. These rates did not change much with the total number of patients.

The relative bias, the standard deviance, and the RMSE of the parameters are shown in Tables A2 to A4. Overall, the relative bias of the parameters was lower than 5%, except for parameters k_{12}, k_{21}, and A_2. In fact, parameters k_{12} and k_{21} are linked and difficult to estimate from the data because there were no measurements in Compartment 2. Hence, in any simulation, an overestimation of k_{12} may be compensated by an underestimation of k_{21} and conversely. It is consequently difficult to analyze separately the relative biases of these two parameters. Generally, the absolute relative bias increases with the decrease in total number of patients and with the increase of the residual variance. The RMSE was generally low, except for k_{12} and k_{21}.

In conclusion, the global classification error rate was acceptable (close to 7%) for realistic residual variances and the relative biases were generally acceptable (lower than 5%). Given these results, when ordinary differential equations are used to model the trajectories, the algorithm we propose to identify the groups and estimate the group parameters seems appropriate.

Table A1. Global classification error rates and per-group classification error rates according to the simulation parameters.

Simulation parameter				Classification error rate			
N_1	N_2	N_3	σ	Global	In group 1	In group 2	In group 3
100	500	400	0.747	0.068	0.130	0.050	0.076
20	100	80	0.747	0.069	0.123	0.051	0.078
10	50	40	0.747	0.073	0.132	0.048	0.091
100	500	400	1.121	0.167	0.316	0.114	0.196
20	100	80	1.121	0.174	0.286	0.135	0.194
10	50	40	1.121	0.171	0.262	0.120	0.211

N_1, N_2, N_3: Number of patients per group - σ: Residual variance.

Table A2. Relative biases of the estimated parameters according to the simulation parameters.

Simulation parameter				Relative bias									
N_1	N_2	N_3	σ	k_{12}	k_{21}	k_{10}	$\mu_{01,1}$	$\mu_{01,2}$	$\mu_{01,3}$	A_1	A_2	A_3	σ
100	500	400	0.747	0.531	0.839	-0.004	0.003	-0.001	0.003	0.000	-0.035	-0.006	-0.027
20	100	80	0.747	1.725	2.043	-0.028	0.004	-0.001	0.004	-0.016	-0.159	-0.052	-0.021
10	50	40	0.747	1.665	2.879	-0.045	0.005	-0.003	0.003	-0.040	-0.236	-0.069	-0.025
100	500	400	1.121	0.482	0.625	-0.023	0.004	-0.006	0.014	0.021	-0.189	0.000	-0.051
20	100	80	1.121	5.585	7.342	-0.046	0.011	-0.007	0.015	-0.002	-0.329	-0.053	-0.048
10	50	40	1.121	3.937	5.175	-0.058	0.012	-0.008	0.013	-0.034	-0.377	-0.070	-0.058

N_1, N_2, N_3: Number of patients per group - σ: Residual variance - k_{12}, k_{21}, k_{10}: Parameters of the pharmacokinetic model - $\mu_{01,1}$, $\mu_{01,2}$, $\mu_{01,3}$: Initial hCG concentration in the different groups - A_1, A_2, A_3: Constant for the residual hCG production over time in the different groups.

Table A3. Standard deviations of the estimated parameters according to the simulation parameters.

Simulation parameter				Standard deviation									
N_1	N_2	N_3	σ	k_{12}	k_{21}	k_{10}	$\mu_{01,1}$	$\mu_{01,2}$	$\mu_{01,3}$	A_1	A_2	A_3	σ
100	500	400	0.747	0.234	6.590	0.002	0.112	0.058	0.059	0.001	0.001	0.001	0.010
20	100	80	0.747	0.254	5.167	0.004	0.192	0.113	0.138	0.003	0.002	0.002	0.022
10	50	40	0.747	0.229	7.901	0.006	0.319	0.179	0.177	0.004	0.003	0.003	0.026
100	500	400	1.121	0.081	2.115	0.003	0.234	0.118	0.128	0.002	0.002	0.002	0.017
20	100	80	1.121	1.157	28.600	0.006	0.400	0.216	0.270	0.004	0.002	0.003	0.039
10	50	40	1.121	0.479	13.069	0.006	0.562	0.290	0.323	0.005	0.003	0.004	0.039

N_1, N_2, N_3: Number of patients per group - σ: Residual variance - k_{12}, k_{21}, k_{10}: Parameters of the pharmacokinetic model - $\mu_{01,1}$, $\mu_{01,2}$, $\mu_{01,3}$: Initial hCG concentration in the different groups - A_1, A_2, A_3: Constant for the residual hCG production over time in the different groups.

Table A4. RMSEs of the estimated parameters according to the simulation parameters.

Simulation parameter				RMSE									
N_1	N_2	N_3	σ	k_{12}	k_{21}	k_{10}	$\mu_{01,1}$	$\mu_{01,2}$	$\mu_{01,3}$	A_1	A_2	A_3	σ
100	500	400	0.747	0.055	43.621	0.000	0.014	0.004	0.005	0.000	0.000	0.000	0.001
20	100	80	0.747	0.073	30.159	0.000	0.040	0.013	0.022	0.000	0.000	0.000	0.001
10	50	40	0.747	0.061	69.211	0.000	0.104	0.033	0.032	0.000	0.000	0.000	0.001
100	500	400	1.121	0.007	4.778	0.000	0.057	0.019	0.047	0.000	0.000	0.000	0.003
20	100	80	1.121	1.426	857.955	0.000	0.179	0.053	0.109	0.000	0.000	0.000	0.004
10	50	40	1.121	0.278	193.006	0.000	0.337	0.091	0.131	0.000	0.000	0.000	0.006

RMSE: Root mean squared error - N_1, N_2, N_3: Patient number per group - σ: Residual variance - k_{12}, k_{21}, k_{10}: Parameters of the pharmacokinetic model - $\mu_{01,1}$, $\mu_{01,2}$, $\mu_{01,3}$: Initial hCG concentration in the different groups - A_1, A_2, A_3: Constant for the residual hCG production over time in the different groups.
